# Short-Term Outcomes of Post-Mastectomy Immediate Pre-Pectoral Reconstruction with Implant and Acellular Dermal Matrix

**DOI:** 10.3390/jcm14207181

**Published:** 2025-10-11

**Authors:** Beatriz Costeira, Beatriz Gonçalves, António Soares, Rodrigo Oom, Cristina Sousa Costa, João Vargas Moniz, Nuno Abecasis, Catarina Rodrigues dos Santos

**Affiliations:** 1 Breast Clinic, Instituto Português de Oncologia de Lisboa Francisco Gentil (IPOLFG), R. Prof. Lima Basto, 1099-023 Lisboa, Portugal; bgoncalves@ipolisboa.min-saude.pt (B.G.); amsoares@ipolisboa.min-saude.pt (A.S.); room@ipolisboa.min-saude.pt (R.O.); cscosta@ipolisboa.min-saude.pt (C.S.C.); jmoniz@ipolisboa.min-saude.pt (J.V.M.); 2 Department of General Surgery, Instituto Português de Oncologia de Lisboa Francisco Gentil (IPOLFG), R. Prof. Lima Basto, 1099-023 Lisboa, Portugal; nabecasis@ipolisboa.min-saude.pt; 3 Faculdade de Medicina da Universidade de Lisboa, Av. Prof. Egas Moniz, 1649-028 Lisboa, Portugal; catarinarsantos@hotmail.com

**Keywords:** breast cancer, mastectomy, post-mastectomy immediate reconstruction, direct-to-implant, acellular dermal matrix

## Abstract

**Introduction:** Pre-pectoral breast reconstruction using implant and acellular dermal matrix (ADM) has become one of the main techniques for immediate reconstruction after mastectomy, with variable approaches and complication rates reported in literature. This study aims to evaluate the early outcomes of this technique, at a single tertiary oncology center. **Methods:** We performed a retrospective analysis of a prospectively maintained database including women who underwent immediate pre-pectoral reconstruction with implant and ADM following mastectomy between January 2021 and August 2023. The primary outcome was reconstructive failure within 3 months, defined as the need for removal of the implant placed during the index surgery. Secondary outcomes included complications at 3 months and predictive factors for complications. **Results:** A total of 247 reconstructions were performed in 200 patients, 83.4% following oncological mastectomy and 16.6% after risk-reducing surgery. The median age was 49 (43–56) years; 15.5% of patients were obese and 26.5% were active smokers. Skin-sparing mastectomy was performed in 16.6% and nipple-sparing in 83.4%, with a Wise-pattern incision in 73.3%. Reconstructive failure occurred in 7.7%, with one case (0.4%) of total reconstruction loss. The overall complication rate was 14.6%—skin flap ischemia occurred in 12.6%, primary implant infection in 2.0% and bleeding in 0.8%. The reoperation rate was 8.4%. No predictive factors for complications were identified. **Conclusions:** In this series, including a high proportion of high-risk patients, immediate pre-pectoral reconstruction with implant and ADM appears safe, with a low rate of early complications. No predictive factors for complications were found, supporting widening its indications.

## 1. Introduction

Breast cancer is the leading cancer among women worldwide [1], and surgery remains a central step in its curative treatment. Despite the advances in systemic therapies and the possibility of surgical de-escalation, mastectomy is still required in approximately 40% of cases [2,3]. This may be partly due to the increasing use of magnetic resonance imaging (MRI), with consequent increased diagnosis of multicentric tumors, as well as the growing awareness of genetic risk factors prompting risk-reducing mastectomies [3,4].

Skin- and nipple-sparing mastectomies have been proven oncologically safe [5,6,7] and allow for immediate breast reconstruction, which some studies suggest is associated with improved quality of life [8,9]. In this setting, implant-based reconstruction (IBR) is the most commonly used technique [10,11], whether using tissue expanders or a direct-to-implant (DTI) approach. Traditionally, implants were placed in a submuscular pocket created beneath the pectoralis major muscle. More recently, the introduction of materials such as acellular dermal matrix (ADM), offering pocket control and implant support, has led to an increased adoption of DTI techniques and a switch to pre-pectoral reconstruction [12].

Although most evidence regarding ADM use stems from retrospective studies, it appears to be associated with lower rates of device exposure and capsular contracture, along with improved aesthetic outcomes [13]. A recent meta-analysis also showed a trend toward lower rates of reconstructive failure, seroma, hematoma, flap ischemia/necrosis, and late rippling with ADM-based pre-pectoral reconstruction, though differences were not statistically significant [14]. Notably, none of the included studies adjusted for surgeon selection bias, as ADM is often preferred in thinner or less viable flaps, potentially confounding results. Similarly, evidence on implant topography remains limited and influenced by institutional protocols and surgeon preference. Available data show comparable rates of early complications (skin necrosis or infection) between pre- and retro-pectoral reconstructions, but the retro-pectoral approach is linked to greater postoperative pain, reduced arm mobility, animation deformity, and insufficient lower pole fullness, contributing to higher rates of prosthesis failure [15,16,17].

Standard risk factors for IBR include diabetes, obesity, smoking, and prior radiotherapy [11,18]. Initially, concerns regarding the safety of pre-pectoral DTI in these populations led to their exclusion from most studies. However, improved control of certain comorbidities (e.g., diabetes), alongside rising rates of others (e.g., obesity), has led to the broader inclusion of high-risk patients in clinical practice for immediate pre-pectoral IBR [19,20].

At our institution, a tertiary oncology referral center, pre-pectoral implant-based reconstruction with ADM was first introduced in 2021 and gradually expanded to patients with higher-risk profiles. Given the limited evidence on short-term outcomes —particularly in such populations—this study aims to assess the early postoperative results of this technique in a real-world oncologic setting.

## 2. Materials and Methods

This was a retrospective, single-center cohort study including women who underwent mastectomy with immediate pre-pectoral implant-based reconstruction using an ADM between January 2021 and August 2023.

Ethics committee approval was not required for this retrospective single-center analysis.

All patients underwent a preoperative workup consisting of clinical assessment, bilateral breast ultrasonography, mammography, magnetic resonance imaging, and tumor biopsy when indicated. Systemic staging was performed in all patients with breast cancer staged ≥cT2 or cN+ [21]. Treatment strategy for each patient was determined at a dedicated multidisciplinary breast cancer meeting, in accordance with institutional protocols. Genetic risk counseling was offered to patients diagnosed with breast cancer before the age of 50, those with bilateral disease, a personal history of other malignancies, a strong family history of cancer, or known familial pathogenic variants.

Immediate breast reconstruction following mastectomy was offered to all patients in whom the skin, with or without the nipple-areolar complex (NAC), could be preserved. Contraindications for immediate breast reconstruction included inflammatory breast cancer and patient preference. Contraindications for pre-pectoral DTI reconstruction included prior radiotherapy and patient preference for autologous reconstruction.

All surgeries were performed by one of four breast-dedicated surgeons from the institutional team. A nipple-sparing mastectomy (NSM) was offered whenever preoperative imaging excluded NAC involvement; otherwise, a skin-sparing mastectomy (SSM) was planned. For patients scheduled for NSM, intraoperative histologic assessment of retroareolar tissue was routinely performed. If positive for malignancy, the NAC was excised and the procedure converted to an SSM. For small to medium-sized breasts with normal or grade I ptosis, according to Regnault’s classification [22], NSM was performed through an inframammary incision, whereas SSM employed an elliptical incision including the NAC. For medium to large-sized breasts or those with grade II–III ptosis [22], a Wise-pattern incision was preferred for both NSM and SSM, using a de-epithelialized inferior dermal flap. In NSM cases requiring skin reduction, NAC preservation was achieved either through a superior pedicle technique or free nipple grafting, at the discretion of the breast surgeon.

A sheet of porcine-derived ADM (CELLIS^®^ Breast - Meccellis Biotech, La Rochelle, France) was brought to the surgical field and placed in a saline bath. All implants used were silicone gel, textured, anatomic implants (Mentor^® -^ Mentor Worldwide LLC, CA, USA). On the back table, the ADM was fenestrated and secured around the implant with absorbable sutures, ensuring complete coverage. The ADM-covered implant was then placed into the subcutaneous pocket and anchored to the pectoralis major muscle using absorbable sutures. For patients undergoing unilateral mastectomy, a contralateral procedure was performed when necessary to achieve breast symmetry.

One or two suction drains were placed: one in the mastectomy pocket and a second in the axilla, if axillary lymph node dissection was performed. Drains were removed once 24 h output decreased to <50 mL. Prophylactic antibiotic therapy consisted of an intraoperative dose of intravenous cefazolin (1 g), repeated every two hours during surgery, followed by a seven-day course of oral cefradine (500 mg every 12 h). All patients were instructed to wear a surgical compression bra for two months postoperatively.

Postoperative follow-up after hospital discharge included weekly visits with a breast-dedicated nurse until all wounds had healed and drains had been removed, as well as scheduled medical appointments at 1 and 3 months, with additional visits on demand in between.

Data collected included patient demographics, tumor characteristics, surgical details, and 90-day postoperative follow-up.

The primary outcome was reconstructive failure within 90 days postoperatively, defined as the need for removal of the implant placed during the index surgery, with or without subsequent reconstruction using an autologous flap or a new implant/tissue expander. Secondary outcomes included 90-day complication rates—specifically, skin flap ischemia (superficial or full-thickness, including wound dehiscence and NAC ischemia in NSM), infection, bleeding, reoperation—as well as potential predictive factors for these complications.

Statistical analysis was performed using IBM SPSS Statistics, version 26.0 (IBM Corporation, Armonk, NY, USA). Continuous variables were reported as medians and interquartile range (25–75th percentile), and categorical variables as absolute value and frequency. Continuous variables were compared using Student’s t-test, and categorical variables using the chi-square test. A logistic regression model, including both univariate and multivariate analyses, was used to identify predictive factors associated with complications. Only variables found to be significant in the univariate analysis were included in the multivariate regression model. A two-tailed *p* value <0.05 was considered statistically significant.

## 3. Results

A total of 247 reconstructions in 200 female patients were included, with a median age of 49 (43;56) years. The median body mass index (BMI) was 26 (23;29) kg/m^2^, with 56.5% of patients being overweight (BMI 25–29.9 kg/m^2^) and 15.5% obese (BMI ≥ 30 kg/m^2^). At the time of surgery, 26.5% were active smokers. A pathogenic germline mutation associated with increased breast cancer risk was identified in 21% of patients, most commonly BRCA2 (11%).

The most frequent indication for mastectomy was invasive breast cancer (70.9%), followed by risk-reducing/prophylactic surgery (18.6%) and ductal carcinoma in situ (10.5%). Overall, 81.4% of procedures were performed for oncological indication. Neoadjuvant chemotherapy was administered to 45.7% of patients.

Among patients with invasive breast cancer (n = 175), most tumors were staged as cT2 (50.9%), and 35.4% were node positive. Of the 65 tumors staged as cT1 (37.1%), 73.8% (48/65) were multicentric or associated with extensive in situ disease precluding breast conservative surgery (BCS), and 23.1% (15/65) were diagnosed in patients carrying a germline mutation. Histologically, most tumors were non-special type (82.3%) and in terms of molecular subtypes, most were luminal A (40.0%) and luminal B/HER2-negative (37.1%). Patient and tumor characteristics are summarized in Table 1.

NSM was performed in 83.4% of cases and SSM in 16.6%. In the NSM group (n = 206), the most common incision was the Wise pattern (73.3%) and free grafting was used for NAC preservation in 40.3%. In the SSM group (n = 41), the Wise pattern was also the most frequently used incision (83.0%). Implant size ranged from 125 to 650 cc, with a median of 375 (330;475) cc. Bilateral surgery was performed in 90.5%, most commonly contralateral symmetrization.

**Figure 1:**
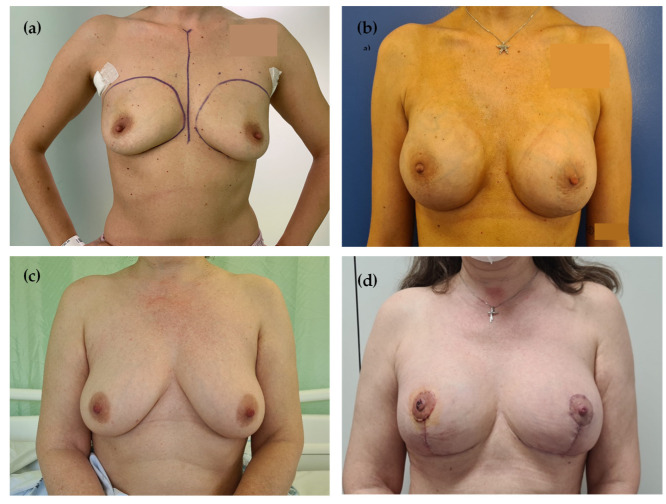
Before (**a**) and after (**b**) bilateral NSM; Before (**c**) and after (**d**) right NSM and contralateral mammoplasty.

Surgical details are summarized in Table 2. Representative pre- and postoperative outcomes are shown in Figure 1a–d.

The overall 90-day complication rate was 14.6%. The most frequent complication was skin ischemia (12.6%), followed by primary implant infection (2.0%) and hemorrhage (0.8%). Two patients develop two simultaneous complications (hemorrhage and NAC ischemia; infection and NAC ischemia).

The reoperation rate at 90 days was 8.4%. The majority of reoperations (82.6%) were due to reconstructive failure. Other indications included bleeding (8.7%), scar revision (4.3%), and skin grafting (4.3%).

**Figure 2:**
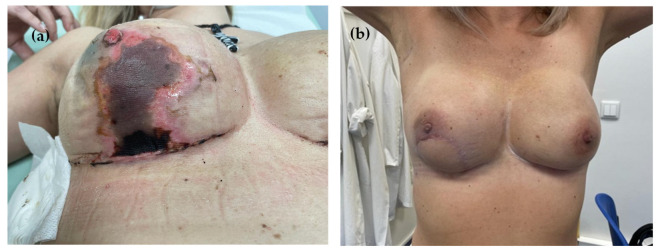
Skin necrosis (**a**) and outcome after reoperation with latissimus dorsi flap (**b**).

Reconstructive failure occurred in 7.7% of cases, most commonly in the context of skin ischemia (54.8% of patients with this complication). Surgical revision required a latissimus dorsi myocutaneous flap in 5.7% of patients (Figure 2a,b); in 1.2%, the implant was replaced by a tissue expander, and in 0.8% by a smaller implant. Complete loss of reconstruction was reported in one patient (0.4%).

In the NSM group (n = 206), NAC loss due to necrosis occurred in 6.8% of cases, with no significant difference between preservation techniques (6.5% for superior pedicle vs. 7.2% for free grafting; *p* = 0.839). In addition, 3 NACs were excised following detection of tumor cells in the final pathological analysis.

Postoperative outcomes are summarized in Table 3.

On univariate analysis, no individual factors (age, comorbidities, oncologic diagnosis) or surgical factors (bilateral surgery, mastectomy type, Wise-pattern incision, implant volume) were found to be significantly associated with postoperative complications, including skin ischemia or reoperation. Risk factors for hemorrhage and infection were not assessed due to their low incidence. A subgroup analysis of patients undergoing NSM also failed to identify any predictors of NAC loss. Since no variables were associated with outcomes on univariate analysis, multivariate regression was not performed. The univariate logistic regression model is presented in Table 4.

## 4. Discussion

We report a series of 247 mastectomies with immediate pre-pectoral implant–ADM reconstruction performed over a 2.5-year period, the majority in patients with breast cancer and including a substantial proportion of smokers, overweight or obese patients, and Wise-pattern incisions. We achieved a reconstructive failure rate of 7.7% and an overall 90-day complication rate of 14.6%. This series reflects the implementation of this technique in a breast-dedicated surgical unit treating more than 800 cancer cases annually.

Our primary outcome—reconstructive failure—is frequently overlooked in the literature but represents a clinically important measure of success. Kalstrup et al. [23] retrospectively analyzed 232 pre-pectoral implant–ADM reconstructions and observed a 13.0% explantation rate, including 6.0% complete reconstruction loss. In the RCT by Lohmander et al. [24], comparing dual-plane implant–ADM with retropectoral reconstruction, implant loss occurred in 6.0% of patients in both groups. More recently, in a retrospective case-matched comparison by Kilmer et al., rates of implant loss of 8.2% and 6.3% (ADM and no-ADM) were obtained [25]. Our failure rate of 7.7%, with only one case (0.4%) of complete reconstruction loss, is consistent with all these reports. Reconstruction loss has been associated with adverse effects on patients’ quality of life and self-esteem [26]. More importantly, in the oncologic setting, complications must be promptly addressed to avoid delays in adjuvant treatment that might be indicated.

At 90 days, our overall complication rate was 14.6%, which is relatively low for this procedure. Kalstrup [23] and Lohmander [24] reported complication rates at 6 months of 34% and 45%, respectively. A more recent study examining the 90-day outcomes of pre-pectoral tissue expander ADM-based reconstructions, reported complication rates of 20.8% for the ADM cohort [27]. Although most complications occur within the first postoperative month [28], the longer follow-up in the first two series may account for this difference. Moreover, these studies reported seroma in 8–12% of cases. In our experience, early seroma is not only difficult to accurately assess due to the prolonged presence of a drain, but also of limited clinical relevance.

Among the observed complications, skin ischemia was the most frequent, occurring in 12.6% of patients. This rate is consistent with previously reported values in the literature (7.8–12.2%) [23,29,30] and is likely related to the high proportion of oncologic mastectomies in our cohort. Importantly, we adopted a comprehensive definition of skin ischemia, encompassing superficial ischemia (often underreported and traditionally considered of limited clinical significance, yet a potential cause of prolonged wound care by the nursing team), as well as wound dehiscence and NAC ischemia (specific to NSM).

The second most common complication, primary implant infection, occurred in only 2.0% of cases. A recent meta-analysis including 11,988 ADM-assisted implant reconstructions (pre-pectoral, dual-plane, and subpectoral) reported an overall infection rate of 8.9%, and 11.1% specifically for porcine-derived ADMs [31]. In our series, perioperative prophylaxis consisted of a single intraoperative intravenous (IV) dose followed by a 7-day course of oral antibiotics. Although current evidence does not support routine extended antibiotic prophylaxis [32], this practice remains common in daily clinical practice and may have contributed to our low infection rate. Furthermore, the American Society of Plastic Surgeons suggests that the presence of a drain adjacent to the implant may warrant consideration of extended antibiotic prophylaxis [33].

Traditional risk factors for this technique include diabetes, obesity, and active smoking, which have been associated with higher rates of mastectomy flap necrosis, implant infection, and extrusion [34,35,36]. In our cohort, 56.5% of patients were overweight, 15.5% obese, and 26.5% active smokers—a considerably higher proportion of high-risk patients compared with previously published series [19,27,37]. Nevertheless, we observed no increase in complications within these subgroups (*p* > 0.05). These findings align with recent evidence suggesting that outcomes in obese patients are comparable to those in non-obese patients and that BMI should not serve as an exclusion criterion for this technique [31,38,39]. Smoking cessation should always be encouraged; however, smokers may still be considered for implant-based reconstruction when appropriately selected. Also, overweight is often associated with breast ptosis, which may justify the need for skin reduction and may explain our unusually high rate of Wise-pattern mastectomies (74.9%). Although this incision has also been described as high risk in pre-pectoral reconstruction [40,41], we found no association with increased complication rates. In our experience and in parallel with the recent finding of Antoniazzi et al. [42], when performed by experienced teams, this approach can be safely implemented while improving aesthetic outcomes.

In the context of skin reduction, NAC relocation remains a key challenge. In our practice, this can be addressed by combining a reduction pattern with NAC free grafting. Using this method, we preserved 40.3% of NACs. In cases of NAC loss, free skin grafting allowed successful replacement and salvage of the breast reconstruction.

Another relevant aspect is the high proportion of bilateral procedures, performed in 90.5% of patients, including 23.5% bilateral mastectomies, without an associated increase in complications. This strategy may optimize medical and hospital resources, including operating room time and hospital stay, while reducing the number of surgical episodes and minimizing disruption to breast cancer patients’ daily lives.

This study has several limitations. First, it is a retrospective, single-center analysis based on clinical records, with the inherent risk of bias—particularly the potential underreporting of minor complications such as superficial ischemia. Second, long-term follow-up data were not available, especially regarding adjuvant treatments or late complications (e.g., rippling or capsular contracture). Finally, no comparison with other reconstructive techniques was performed, and data on quality of life, patient satisfaction, and economic impact were not assessed. It should also be noted that the low number of events may have limited the statistical power of the logistic regression analysis, and larger series are warranted to confirm these findings.

Nevertheless, we believe that the results of this series contribute to expanding current knowledge on pre-pectoral immediate breast reconstruction with implant and ADM, including in high-risk patients. This report reflects the experience of a dedicated center with a technique that continues to evolve.

## 5. Conclusions

In high-volume centers, immediate pre-pectoral breast reconstruction with an implant and acellular dermal matrix (ADM) appears to be a safe and feasible technique, with low rates of reconstructive failure and early complications, even among high-risk patients. No predictive factors for complications were identified, supporting the potential to broaden its indications. Prospective studies with larger cohorts and extended follow-up are needed to strengthen these findings.

## Figures and Tables

**Table 1:** Patient’s and tumor’s characteristics.

	N = 200
Age in years, median [IQR]	49 (43;56)
BMI	
Median [IQR], kg/m2	26 (23;29)
Overweight (BMI 25–29.9 kg/m^2^), n (%)	113 (56.5)
Obese (BMI > 30 kg/m^2^), n (%)	31(15.5)
Active smoker, n (%)	53 (26.5)
Comorbidities, n (%)	
Diabetes mellitus	7 (3.5)
Dyslipidemia	15 (7.5)
Hypertension	7 (3.5)
Genetic risk, n (%)	42 (21.0)
BRCA1	11 (5.5)
BRCA2	22 (11.0)
CHEK2	5 (2.5)
PALB2	3 (1.5)
TP53	1 (0.5)
Bilateral surgery, n (%)	181 (90.5)
Bilateral mastectomy	47 (23.5)
Contralateral BCS	5 (2.5)
Contralateral symmetrization	129 (64.5)
Mastectomy indication, n (%)—n = 247	
Invasive carcinoma	175 (70.9)
In situ carcinoma	26 (10.5)
Risk-reducing	46 (18.6)
Neoadjuvant chemotherapy, n (%)	80 (45.7)
cT staging, n (%)—n = 175	
1	65 (37.1)
2	89 (50.9)
3	21 (12.0)
cN staging, n (%)—n = 175	
0	113 (64.6)
1	57 (32.6)
2	5 (2.8)
Histologic subtypes, n (%)—n = 175	
NST	144 (82.3)
Lobular	26 (14.9)
Metaplastic	2 (1.1)
Mucinous	2 (1.1)
Neuroendocrine	1 (0.6)
Molecular subtypes, n (%)—n = 175	
Luminal A	70 (40.0)
Luminal B Her2−	65 (37.1)
Luminal B Her2+	10 (5.7)
Her2 type	12 (6.9)
Triple negative	17 (9.7)
Missing	1 (0.6)

BCS—breast conservative surgery; BMI—Body mass index; IQR—inter-quartile range; NST—Non-special type.

**Table 2:** Surgery details.

	N = 247
Mastectomy type, n (%)	
Nipple-sparing mastectomy (NSM)	206 (83.4)
Skin-sparing mastectomy (SSM)	41 (16.6)
Mastectomy incision, n (%)	
NSM—n = 206	
Wise-pattern	151 (73.3)
Inframammary	40 (19.4)
Periareolar	8 (3.9)
Lateral	7 (3.4)
SSM—n = 41	
Wise-pattern	34 (83.0)
Periareolar (elliptical)	4 (9.7)
Inframammary (secondary NAC excision)	3 (7.3)
NAC preservation in NSM, n (%)—n = 206	
Superior pedicle	123 (59.7)
Free grafting	83 (40.3)
Implant size (cc), median [IQR]	375 (330;475)
Length of stay (days), median [IQR]	2 (2;2)

IQR—inter-quartile range; NAC—Nipple-areolar complex; NSM—Nipple-sparing mastectomy; SSM—Skin-sparing mastectomy.

**Table 3:** Postoperative complications.

	N = 247
90-day complication, n (%)	36 (14.6)
Type of complication, n (%)	
Skin ischemia/necrosis (including NAC)	31 (12.6)
Primary implant infection	5 (2.0)
Hemorrhage	2 (0.8)
NAC necrosis, n (%)—n = 206	14 (6.8)
90-day re-operation, n (%)	23 (8.4)
Re-operation procedure, n (%)—n = 23	
Latissimus dorsi flap	14 (60.9)
Tissue expander replacement	3 (13.1)
Implant replacement	2 (8.7)
Hemostasis revision	2 (8.7)
Scar revision	1 (4.3)
Skin graft	1 (4.3)
Reconstructive failure, n (%)	19 (7.7)
Complete loss of reconstruction, n (%)	1 (0.4)

NAC—Nipple-areolar complex.

**Table 4:** Univariate risk factor analysis.

Variables	Any Complication		Skin Ischemia		Reoperation		NAC Loss (Subgroup NSM)	
	OR	*p*-Value	OR	*p*-Value	OR	*p*-Value	OR	*p*-Value
Age, years	1.01 (0.98–1.04)	0.616	1.01 (0.97–1.05)	0.639	0.99 (0.96–1.04)	0.925	0.98 (0.94–1.03)	0.407
Obesity (BMI ≥ 30)	1.09 (0.39–3.07)	0.872	0.7 (0.20–2.53)	0.601	0.80 (0.22–2.86)	0.729	0.67 (0.15–3.05)	0.599
Overweight (BMI 25–29.9)	1.04 (0.47–2.32)	0.924	1.06 (0.44–2.54)	0.895	0.90 (0.37–2.22)	0.826	0.90 (0.33–2.47)	0.842
Active smoker	0.75 (0.31–1.85)	0.536	1.16 (0.46–2.97)	0.754	0.59 (0.19–1.83)	0.355	1.81 (0.62–5.30)	0.275
Diagnosis (oncologic vs. prophylactic)	0.50 (0.23–1.24)	0.139	0.58 (0.23–1.50)	0.250	0.48 (0.19–1.25)	0.127	0.79 (0.24–2.52)	0.690
Neoadjuvant chemotherapy	0.51 (0.22–1.14)	0.095	0.38 (0.15–0.99)	0.042	0.46 (0.18–1.22)	0.111	0.52 (0.18–1.51)	0.221
Bilateral breast surgery	3.39 (0.43–6.57)	0.218	1.13 (1.13–1.08)	0.143	1.11 (1.06–1.16)	0.171	1.09 (1.05–1.13)	0.230
NSM (vs. SSM)	1.65 (0.55–4.96)	0.369	1.80 (0.52–6.27)	0.351	1.53 (0.44–5.42)	0.500	--	--
Wise-pattern incision	1.30 (0.58–2.94)	0.524	1.49 (0.54–4.15)	0.438	0.48 (0.19–1.25)	0.127	0.831 (0.28–2.51)	0.743
Implant volume	1.00 (0.99–1.01)	0.785	1.00 (0.99–1.01)	0.661	1.00 (0.99–1.01)	0.796	1.00 (0.99–1.01)	0.507

BMI—Body mass index; NAC—Nipple-areolar complex; NSM—Nipple-sparing mastectomy; OR—Odds ratio; SSM—Skin-sparing mastectomy.

## Data Availability

The data presented in this study are available on request from the corresponding authors due to ethical reasons.

## Abbreviations

The following abbreviations are used in this manuscript:

ADM	Acellular Dermal Matrix
BCS	Breast Conservative Surgery
BMI	Body Mass Index
DTI	Direct-to-Implant
IBR	Implant-based Reconstruction
IQR	Inter-quartile Range
MRI	Magnetic Resonance Imaging
NAC	Nipple-areolar Complex
NSM	Nipple-sparing Mastectomy
NST	Non-special Type
SSM	Skin-sparing Mastectomy
